# Are born global firms really a “new breed” of exporters? Empirical evidence from an emerging market

**DOI:** 10.1007/s10843-022-00307-0

**Published:** 2022-01-13

**Authors:** Øystein Moen, Mohammad Falahat, Yan-Yin Lee

**Affiliations:** 1 Department of Industrial Economics and Technology Management, Norwegian University of Science and Technology, Alfred Getz vei 3, 7491 Trondheim, Norway; 2 Universiti Tunku Abdul Rahman (UTAR), Bandar Sungai Long, 43000 Kajang, Selangor Malaysia

**Keywords:** Strategic orientation, Marketing capabilities, International performance, Digital orientation, Governmental support

## Abstract

This study investigates and compares born global (BG) firms and non-BG firms in Malaysia. We employed the multigroup analysis technique with structural equation models to test six hypotheses to determine the differences and similarities between two proposed models of BG and non-BG firms across a wide range of industries. The study reveals differences between the antecedents of marketing capabilities for BGs versus non-BGs, indicating that the performance enabling mechanisms differ between the groups. More precisely, the ability of BG firms to convert digital and entrepreneurial orientations into marketing capabilities is found to be a distinguishing characteristic of these firms. Moreover, non-BGs utilize government support to build marketing capabilities and obtain superior performance in the international market. This result suggests that governmental export promotion initiatives in Malaysia should be adjusted to increase relevance for BGs. The findings indicate that marketing capabilities play an essential role in the international market performance of both BGs and non-BGs. An important implication is that managerial focus and actions need to be adjusted depending on the type of firm. The two types of firms are not equal; if they are managed similarly, misjudgment will arise.

## Abstract (in German)

Diese Studie untersucht und vergleicht Born-Global-Unternehmen (BG) und Nicht-Born-Global-Unternehmen (Nicht-BG) in Malaysia. Die Autoren verwendeten die Multi Criteria Analysis (multikriterielle Entscheidungsanalyse) von Strukturgleichungsmodellen, um Hypothesen zur Bestimmung von Unterschieden und Ähnlichkeiten zwischen zwei vorgeschlagenen Modellen von BGs und Nicht-BGs in einer Vielzahl von Branchen zu testen. Die Studie zeigt Unterschiede zwischen den Vorläufern der Vermarktungsfähigkeiten für BGs und Nicht-BGs auf, was darauf hindeutet, dass die leistungsfördernden Mechanismen zwischen den Gruppen unterschiedlich sind. Genauer gesagt: Die Fähigkeit von BGs, digitale und unternehmerische Orientierungen in Vermarktungsfähigkeiten zu überführen, erweist sich als unterscheidendes Merkmal dieser Unternehmen. Die Ergebnisse zeigten auch, dass Nicht-BGs staatliche Unterstützung nutzen, um Vermarktungskapazitäten aufzubauen und eine überlegene Leistung auf dem internationalen Markt zu erzielen. Dieses Ergebnis legt nahe, dass staatliche Exportförderungsinitiativen in Malaysia angepasst werden sollten, um für BGs relevanter zu sein. Die Ergebnisse deuten darauf hin, dass Vermarktungsfähigkeiten eine wesentliche Rolle für die Leistung sowohl von BGs als auch von Nicht-BGs auf dem internationalen Markt spielen. Eine wichtige Schlussfolgerung ist, dass der Fokus und die Maßnahmen des Managements je nach Unternehmensart angepasst werden müssen. Die beiden Unternehmensarten sind nicht gleich; wenn sie gleichbehandelt werden, führt dies zu Fehleinschätzungen.

## Summary highlights

*Contributions*: Since the inception of the literature focusing on born global (BG) firms, an important question has been how different these firms are from other exporting firms. We present an empirical study investigating how BG transforms different managerial orientations into marketing capabilities and international performance and compare this to what we find for non-BG firms.

*Research questions and purpose*: We include five orientations (digital, entrepreneurial, learning, market, and governmental support orientation) and examine the impact on market capabilities and international performance among BG and non-BG firms.

*Basic research questions, theory, or conceptual framework*: In our hypotheses, we suggest that BG firms can better use digital, entrepreneurial, learning, and market orientations to develop marketing capabilities. We did not expect differences related to governmental support. We further expected BG firms to have a better ability to use marketing capabilities to improve international performance.

*Basic research methodology and information/data*: We conduct multiple group analysis with partial least squares structural equation modeling (MGA PLS-SEM) to test our model exploring differences in the mechanism across the two groups. The data are a sample with 196 valid responses from exporting firms in Malaysia.

*Results/findings*: When examining the digital orientation and entrepreneurial orientation, we found that BG firms were significantly better at building marketing capabilities based on these two orientations. These differences suggest that BG firms and non-BG firms fundamentally differ.

*Limitations*: Our study examined data from a single country, which represents a limitation of the results. In addition, we asked the respondents to focus on the most important product in the most important international market, which may have influenced the results.

*Theoretical implications and recommendations*: We focused on the question of how different BG firms are from other exporting firms, and we observed key differences in how they are able to build marketing capabilities based on digital and entrepreneurial orientations.

*Practical implications and recommendations*: An important implication is that managerial focus and actions need to be adjusted depending on the type of firm. The two groups of firms are not equal; if they are managed similarly, this would result in misjudgment.

*Policy recommendations*: The results also suggest that non-BG firms in Malaysia do benefit more from governmental export orientation than BG firms. This result may indicate the need to further develop governmental export support initiatives to benefit both groups of firms.

*Suggestions for future research directions*: Our results suggest that we should design further research and collect data to improve our understanding of the differences between BG firms and other exporting firms.

## Introduction

Interest in young firms with significant international involvement has been growing, with labels such as born globals **(**BGs) **(**Rennie 1993), international new ventures **(**McDougall et al. 1994), or global start-ups **(**Jolly et al. 1992). Rennie (1993) described these firms as a new breed of exporters based on the difference between them and other exporting firms. The focus on BG firms in recent decades has been driven by their importance for growth in export revenues and employment, as described by Moen and Rialp (2018). Cavusgil and Knight (2015) stated that empirical studies from around the world show that BG firms contribute to a substantial share of export growth.

It is well documented that a limited number of firms achieve significant growth and that BG-type firms constitute an important group of high-growth young firms **(**Choquette et al. 2016). Sleuwaegen and Onkelinx (2014) concluded that BG firms have had the highest rates of both growth and failure, while the importance of these firms and the need for public policy initiatives supporting them have been discussed in both Organisation for Economic Co-operation and Development publications **(**OECD 2013) and reports from the European Union **(**Eurofound 2012). In this paper, we focus on the speed of internationalization, defining BGs as firms with significant international activities within 3 years from inception, following the examples of Falahat et al. (2020); Falahat et al. (2018); Gerschewski et al. (2016); and Gonzalez-Perez et al. (2016). Initially, we would also include export share as criteria, but missing values in the dataset made this difficult, as will also be commented on in the “Methods” section.

McDougall (1989) concluded that there are significant differences between new firms competing in the home market and new firms entering international markets. Differences may also exist between groups of internationally oriented firms, and international entrepreneurship scholars generally agree on the behavioral differences between BG and non-BG firms in terms of the former’s precocity and speed in achieving an international market presence **(**Dimitratos et al. 2016; Falahat and Migin 2017). Some comparative case studies demonstrate the difference between BG and non-BG firms in their abilities to pursue and achieve international success **(**Langseth et al. 2016; Rialp et al. 2005). Gerschewski et al. (2015) pointed out that a comparative approach is necessary to distinguish the antecedents of BGs’ international performance from the variety of antecedents that factor into all firms’ international performance.

Although the research stream focusing on BGs is more than two decades old **(**Rennie 1993), there remains a lack of empirical evidence on the determinants of international performance for these firms compared to other firms with international activities **(**e.g., Andersson et al. 2006; Braunerhjelm and Halldin 2019; Paul and Rosado-Serrano 2019).

This study investigates how five orientations **(**entrepreneurial orientation, learning orientation, market orientation, digital orientation, and orientation toward governmental export support initiatives) influence marketing capabilities within a group of BG firms versus a group of non-BG firms. We also assess the interaction between marketing capabilities and international performance by considering two foreign market conditions as control variables: competitive intensity and market turbulence. In addition, company size and industrial sector are included as control variables.

We highlight the contribution of our study based on three important elements. First, it is well established that BG firms in general are often characterized by digital competence and focus (Reuber and Fischer 2011). Our first contribution is to address whether BG firms are more able to transform digital orientation into marketing capabilities than non-BGs are. In the period of the COVID-19 pandemic with travel and contact restrictions, the digital orientation of firms may be even more important than before, supporting the importance of this particular focus. Our second contribution is the inclusion of governmental support orientation and how this may influence marketing capability development differently between the two groups of firms. We expect employment growth to be an increasingly important public policy goal in a situation with high unemployment levels in many countries. The effectiveness of governmental support initiatives in general as well as how they may contribute among BG firms with high growth potential is therefore a key issue. In combination, comparing differences between BG firms and other firms by considering managers’ digital and government support orientations and how these influences marketing capabilities represent a novel approach that can elucidate the similarities and differences between these two groups of international firms.

Third, Evers et al. (2019) highlight the importance of studies focusing on the interaction between strategic orientations and other factors. We do investigate this by focusing on the possible difference in international performance determinants between BGs and non-BGs. BG firms may not only have different characteristics than other international firms, as presented by Moen and Servais (2002), but also have a different pattern of orientations and capabilities. Inspired by a number of comparative studies (Gerschewski et al. 2015; Hennart 2014; Langseth et al. 2016; McDougall 1989; Paul and Rosado-Serrano 2019; Rialp et al. 2005), we contribute empirically based results related to the possible different orientation-capability-performance linkages for BGs compared to non-BGs.

In this study, we utilize multiple group analysis with partial least squares structural equation modeling **(**MGA PLS-SEM), as proposed by Henseler et al. (2009). This technique is considered suitable to assess direct and indirect relationships and compare BGs to non-BGs **(**Sarstedt et al. 2011).

In the following section, we contextualize the research, develop hypotheses, and present a model that distinguishes the impact of specific antecedents of marketing capabilities and international performance on BG versus non-BG firms.

## Literature review

### Theoretical background

Our overall proposed framework is built on the resource-based view **(**Barney 1991; Barney et al. 2011) and the dynamic capabilities approach **(**Morgan et al. 2018; Teece et al. 1997) to shed light on the crucial role of firm-specific orientations that lead to capabilities for achieving superior international performance. Grounded in institutional theory, we focus on government support as an important dimension of institutional capital that influences a firm’s capabilities (Lu et al. 2010; Oliver 1997). We also embrace the contingency approach used in industrial organization theory **(**Porter 1980) to explain the impact of foreign market conditions on firms’ performance.

We define marketing capabilities as a firm’s ability to leverage the available resources required to perform its marketing tasks and achieve its desired marketing outcomes **(**Day 1994; Morgan et al. 2012). Such capabilities develop and become integrated in an organization over time and are often described as difficult for competitors to analyze and understand, difficult to imitate, and difficult to substitute. They represent complex knowledge and skills integrated in the processes and routines of an organization, as exemplified by Tan and Sousa (2015), who identified that marketing capabilities have a positive impact on firm performance in international markets.

Morgan et al. (2018) observed that few studies focus on the antecedents, mediators, and moderators of marketing capabilities in an international marketing context. To date, the common drivers of rapid internationalization—such as digital orientation, entrepreneurial orientation, learning orientation, and government support—have not been extensively examined as antecedents of marketing capabilities, especially when comparing BGs versus non-BGs (Paul and Rosado-Serrano 2019). It is important to improve our understanding of how young, small firms with resource constraints develop international marketing capabilities in international markets **(**Morgan et al. 2018) and how they differ from traditional firms in terms of capabilities that contribute to the speed of internationalization.

## Hypotheses

### Digital orientation

Digital orientation refers to a firm’s digital-technology focus and its awareness, adoption, and application of digital technology in its business processes **(**Habibi et al. 2015). Day (1994) noted that the creative use of information technology enhances the marketing capabilities of market-driven organizations. In today’s digital economy, firms leverage digital technology to enhance their marketing efficiency **(**Gregory et al. 2017).

Innovative firms that actively use social media and big data have better business performance **(**Bouwman et al. 2018), and digital orientation also improves “information quality” and “service convenience” **(**Foroudi et al. 2017). In general, the impact of digital technology on marketing capabilities can manifest through more effective and efficient management processes, for example, in managing resources and relationships **(**Pagani and Pardo 2017).

SMEs (small and medium-sized enterprises) that are digitally oriented are more likely to embrace digital technology and reap the benefits of enhanced marketing performance (Westerlund 2020). Digital tools such as big data, social media, and e-commerce provide SMEs with an opportunity to leverage their limited resources for cross-border marketing through stronger marketing capabilities **(**Charoensukmongkol and Tarsakoo 2019; Mikalef et al. 2020; Kim 2020). Despite the many studies on the relationship between a firm’s digital orientation and its marketing outcomes, limited studies have explored the possible differences between BG and non-BG firms in terms of their relationships with digital technology. BGs with limited financial resources rely on the internet for the learning and communication they need to facilitate their rapid entry into the international market **(**Gabrielsson and Manek Kirpalani 2004). Even if they have engaged with distributors, agents, or a multinational corporation for their international sales, the internet is often part of their multiple sales channels **(**Gabrielsson and Gabrielsson 2011). While non-BGs can afford to have wider choices for their marketing activities through traditional or more costly advertising and promotions, we conjecture that BGs, as relatively younger, resource-poor, and more technologically minded firms, have a more distinct ability to exploit digital technology to enhance their marketing capabilities. Hence, we hypothesize as follows:

- H1: The relationship between digital orientation and marketing capabilities is stronger for BG than for non-BG firms.

### Entrepreneurial orientation

Entrepreneurial orientation refers to a firm’s entrepreneurial focus, proactiveness, innovativeness, and risk tolerance in pursuing opportunities and profits from international ventures (Falahat et al. 2018; Knight and Cavusgil 2004). Some studies point to entrepreneurial orientation as one of the key drivers of international performance (Eggers et al. 2017; Rua et al. 2018). Entrepreneurial orientation has also been connected to firms’ marketing capabilities (Lisboa et al. 2011; O’Cass and Ngo 2011). However, these studies do not address whether the firm is a BG or not. In relevant empirical studies, Gerschewski et al. (2015) compared BGs and non-BGs and found that international entrepreneurial orientation was a driver of financial and operational performance when considering BGs. Moreover, Knight and Cavusgil (2004) reported a positive relationship between entrepreneurial orientation and business strategies related to the marketing capabilities of BGs. Discussing and interpreting the study by Knight and Cavusgil (2004), Coviello (2015) suggested that international orientations influence international marketing skills and international performance. Based on a large sample of BG firms, Martin et al. (2020) did find a significant relation between entrepreneurial orientation and marketing capabilities. In Malaysia, Kaur and Sandhu (2014) highlighted entrepreneurial orientation as a critical factor for BG internationalization. Entrepreneurial orientation may be described as a key factor characterizing BG firms, and we conjecture that entrepreneurially oriented BGs concentrate more on developing marketing capabilities and therefore can reach foreign markets earlier and faster than non-BG firms. In conclusion, we expect entrepreneurial orientation not only to be an important antecedent of marketing capabilities (Weerawardena et al. 2007), but also to be more important among BG firms than non-BG firms. Based on the above arguments, the following hypothesis is proposed:

- H2: The relationship between entrepreneurial orientation and marketing capabilities is stronger for BG than for non-BG firms.

### Learning orientation

Learning orientation refers to a firm’s enthusiasm for exploratory or exploitative learning **(**Dutta et al. 2016). A learning-oriented firm is committed to learning from its successes and failures and sharing what it has learned. Marketing capabilities are developed by integrating into the marketing process, acquiring market knowledge, and understanding the market. However, the role of learning orientation in developing marketing capabilities has not been extensively tested **(**Morgan et al. 2018), as scholars have mainly studied the direct relationship between learning orientation and performance **(**Gerschewski et al. 2015; Kocak and Abimbola 2009).

Market-focused learning is suggested to positively affect marketing capabilities in BG firms **(**Weerawardena et al. 2007), as learning orientation helps firms understand market requirements and quickly adapt to market needs. The BG literature reports a strong learning orientation among BGs **(**Kocak and Abimbola 2009; Kropp et al. 2006) and suggests that this is an important driver of these firms’ early internationalizing and international performance (De Clercq et al. 2012). This finding is not surprising, as many BGs are in knowledge-intensive industries and continuous learning is a prerequisite for sustained competitiveness. A BG firm may be characterized as a fast learner, compared with a non-BG, due to its relatively smaller size and simpler organizational structure, which encourage a better flow of knowledge **(**Jantunen et al. 2008). However, in the study of Gerschewski et al. (2015), learning orientation did not directly impact the financial or operational performance of BGs. Liu et al. (2020) suggested that the possible missing link between learning orientation and financial performance could be the relationship between knowledge internalization and international marketing performance. In addition, Cake et al. (2020) found that learning orientation has an impact on radical innovation launch success through marketing capabilities. Ismail and Kuivalainen (2015) observed that firms with international scope and global scope have different levels of internal capabilities; the former relies more on founders’ foreign experiential knowledge, while the latter has heterogeneous capabilities such as dynamic marketing capabilities. Furthermore, Buccieri et al. (2020) found that learning orientation is part of the international entrepreneurial culture that contributes to BGs’ dynamic marketing capabilities in emerging markets. We argue that the difference between BG and non-BG firms could be the extent to which they deploy a learning orientation to enhance their marketing capabilities; this is subsequently reflected in their international performance. Thus, the following hypothesis is proposed:

- H3: The relationship between learning orientation and marketing capabilities is stronger for BG than for non-BG firms.

### Market orientation

Market orientation refers to a firm’s focus on market needs and its sensitivity to changes in the market environment. Market orientation is often an antecedent of marketing capabilities, which have been tested in many international marketing studies (Acikdilli et al., 2020; Alnawas and Farha 2020; Cake et al. 2020), but market orientation in the BG context has yet to receive much attention **(**Øyna and Alon 2018). Relatedly, Morgan et al. (2018) observed that there is insufficient interaction between the academic literature on international business and international marketing in terms of empirical research on BG firms. The positive direct effect of market orientation on firm performance has received strong support **(**Acikdilli et al., 2020; Day 1994; Gerschewski et al. 2015; Gruber-Muecke and Hofer 2015; Jaworski and Kohli 1993; Narver and Slater 1990). However, market orientation could also be regarded as an antecedent of marketing capabilities **(**Alnawas and Farha 2020; Murray et al. 2011). Following the findings of Knight et al. (2004), BGs with a strong market orientation are expected to concentrate on developing the necessary marketing capabilities and are thus able to achieve competitive advantage in foreign markets at a faster pace and with a wider scope. We argue that in order for BG firms to achieve such precocity and speed, the market orientation impact on marketing capabilities must be higher than that of non-BG firms. Hence, we hypothesize as follows:

- H4: The relationship between market orientation and marketing capabilities is stronger for BG than for non-BG firms.

### Government support

Government support refers to government-implemented export-promotion programs that are designed to reduce export barriers and encourage export activities **(**Leonidou et al. 2011; Shamsuddoha et al. 2009). Consequently, scholars argue that the effects of government support on firms’ performance should be linked to one or a few mediators, such as resources and capabilities, or to moderators, such as a commitment to exporting **(**Faroque and Takahashi 2015) or geographical scope (Ismail and Kuivalainen 2015). To date, there are limited empirical studies focusing on the effect of government support on firms’ capabilities **(**Coudounaris 2018). In their comprehensive literature review, Morgan et al. (2018) identified the study by Leonidou et al. (2011) as the only one that tested government support as an antecedent of firms’ marketing capabilities. Leonidou et al. (2011) findings suggested that government support enhances firms’ marketing capabilities and leads to improvements in both the export market and financial performance. With government support, firms are more likely to develop a sound export marketing strategy enabling them to realize their competitive advantages for success in foreign market ventures. Leonidou et al. (2011) findings suggested that the impact of government support is stronger for smaller firms and for firms with less export experience. BGs are typically smaller firms, but compared to other (small) firms, BGs may have more international experience.

Both Bell et al. (2004) and Ojala and Heikkila (2011) state that export promotion organizations often target traditional firms following incremental internationalization processes, with programs or initiatives of less relevance for new, rapidly internationalizing firms. Ojala and Heikkala (2011) also suggested a common challenge with current programs offering general, easily available market information: “and it is seldom the kind of information that is to the benefit of the new growth ventures in their specific, practical problems” (Ojala and Heikkala 2011, page 6).

Overall, we cannot identify a reason suggesting that BGs would be affected by governmental export promotion initiatives differently than other firms would (Buccieri et al. 2020), and we thus propose the following hypothesis:

- H5: The relationship between government support and marketing capabilities is equal for BG and for non-BG firms.

### Marketing capabilities

Marketing capabilities enable firms to explore and exploit the potential of foreign markets **(**Kaleka and Morgan 2017; Leonidou et al. 2011). Marketing capabilities are positively connected to international performance in developed markets **(**Gregory et al. 2017; Kaleka 2011; Kaleka and Morgan 2017; Knight et al. 2004; Leonidou et al. 2011; Morgan et al. 2012; Ripollés and Blesa 2012). As expected, the results have suggested a positive effect on firm performance in emerging markets **(**Khavul et al. 2010; Lu et al. 2010; Murray et al. 2011; O’Cass and Ngo 2011; Pham et al. 2017; Zhou et al. 2012).

In the BG context, marketing capabilities are salient to a firm’s international performance because they help the firm gain a first-mover advantage or realize its competitive advantages in the target market **(**Hartsfield et al. 2008; Knight and Cavusgil 2004; Knight et al. 2004; Weerawardena et al. 2007). Our expectation is that young and flexible BG firms are more able to transfer marketing capabilities for international performance than non-BG firms are, and we therefore propose the following hypothesis:

- H6: The relationship between marketing capabilities and international performance is stronger for BG firms than for non-BG firms.

### Control variables

We have included company size and industrial sector as control variables, in addition to two foreign market conditions, market turbulence and competitive intensity, as these have been identified in the prior literature as fundamental factors influencing firms’ international performance **(**Kaleka 2012; Kaleka and Morgan 2017; Murray et al. 2011). Competitive intensity refers to a firm’s perceptions of the degree of competition in its industry and business environment **(**Jaworski and Kohli 1993; Kaleka and Morgan 2017; Murray et al. 2011). Under high levels of competition, a firm’s international performance is likely to be negatively affected. Research on the effect of competitive intensity on a firm’s performance is not conclusive; some study findings indicated a negative effect **(**Matanda and Freeman 2009; Morgan et al. 2012; Silva et al. 2017), while other studies did not identify a significant effect **(**Ismail and Kuivalainen 2015; Kaleka 2012; Miocevic and Morgan 2018; Morgan et al. 2009). Competition is expected to be unfavorable both to BGs and to non-BG firms because they need to allocate more resources to respond to competitors’ actions and to secure their market share. When comparing these two groups of firms, we do expect BGs to be both highly motivated and competitive, often based on technologically advanced and/or unique products, which may make them more able to handle competitive intensity than non-BG firms are. However, non-BG firms are larger and may possess more resources, which may explain why they may be better able to cope with competitive intensity.

Market turbulence refers to a firm’s perception of the difficulty of predicting customer composition and preferences in its industry and in the business environment **(**Jaworski and Kohli 1993; Murray et al. 2011). The unpredictability of customer demands and preferences means that uncertain market responses to a firm’s products are likely to negatively affect that firm’s international performance **(**Baronchelli and Cassia 2014; Matanda and Freeman 2009). The higher risk of an unfavorable market response under conditions of market turbulence may in general be harmful to young and small BGs with limited resources. High uncertainty in the market may also discourage SMEs from exporting **(**Baronchelli and Cassia 2014). Market turbulence may influence smaller firms, but larger firms may have less ability to adjust, change strategies, and adapt to changes, creating opportunities for BG firms (Andersson et al. 2006). On the other hand, considering the resources that play a significant role in a firm’s ability to withstand market turbulence, non-BGs may be more likely to handle market turbulence that could negatively affect their international performance.

**Fig. 1:**
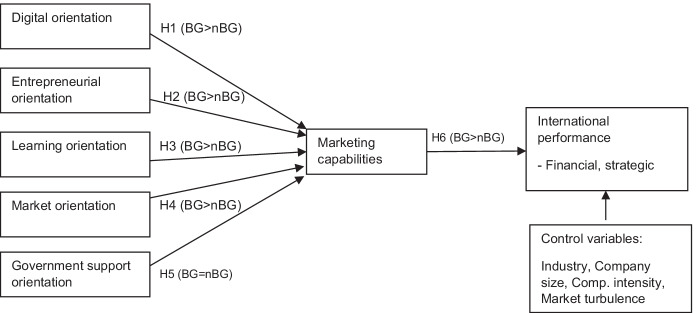
Research model

Based on the above literature, we propose a model, presented in Fig. 1, that focuses on the marketing capabilities that transform critical orientations into international performance. We argue that BG and non-BG firms differ significantly in emerging markets.

## Methods

### Data collection and categorization

We used the Federation of Malaysian Manufacturers **(**FMM) and Malaysian External Trade Development Corporation **(**MATRADE) Directory company address list to identify exporting firms. This is regarded as the most reliable source of exporters in Malaysia. Our sampling frame consists of 1000 companies across all states and industries that had valid contact information and export activities, and we mailed questionnaires. We received 226 responses **(**a 23% response rate) from firms’ founders and senior managers; however, only 196 valid and complete questionnaires were used for this study. A sample size of 196 companies was deemed adequate for this study **(**Cohen 1988; Hair et al. 2014).

We examined the potential nonresponse bias, as per the suggestion of Armstrong and Overton (1977), by comparing the first and last 7% of the surveys we received. The results showed no significant difference between the two groups **(***p* > 0.05). Thus, response bias is unlikely to exist in this study.

We categorized BG firms based on commonly used criteria, namely, exporting within 3 years of inception (Cavusgil, and Knight 2015; Falahat et al. 2018; Gerschewski et al. 2016), whereas non-BG firms start exporting more than 3 years after establishment. Initially, we also wanted to apply a 25% export share requirement (within 3 years), but there were some missing values when measuring export share. Then, we did not use a 25% export share cutoff between the groups. Notably, the average export share was 75% for the BG group and 12% for the non-BG group, suggesting that export involvement differs greatly between these two groups. This actual difference in export shares combined with the threshold of exporting within 3 years is the reason we use the terms BGs and non-BGs to describe the firms in these two groups. In the “Limitations” section, we comment on how our operational definition of BG firms may have influenced the results. On average, the BG firms were 8.7 years old, compared to firms that were 16.5 years old in the non-BG group. The typical BG firm exported to 7 international markets (median value), compared to 5 for the typical non-BG firm. Moreover, the mean number of international markets was 9 among BGs and 12 among non-BGs, and this difference in mean values occurred because of a few non-BGs with a low export share based on a high number of international markets. Our interpretation is that non-BGs seem unable to achieve significant sales volumes in individual export markets. We identified 110 firms within the BG category and 86 firms categorized as non-BGs.

Table 1 summarizes the profiles of these two groups. The BG group is smaller in terms of firm size, and they have a higher percentage of service-oriented firms (27% versus 9%). Assessing industry type, we notice that electronics, electric, machinery, and engineering industries are common among BGs but not among non-BGs.

**Table 1:** Profile of BGs and non-BGs

*Profile of born global and non-born global firms*	*Born global*	*Non-born global*
No. of firms	110	86
Size	Micro and small enterprises	Medium and large enterprises
Average no. of years until exporting	2 years	6 years
Average % of sales from export market	75%	12%
Average age of firm	8.7 years	16.5 years
Number of export markets (median/mean)	7/9	5/12
First export market	Singapore **(**35%) Australia **(**7.3%) UAE **(**6.4%) China **(**5.5%) Thailand **(**5.5%) UK **(**4.5%) Middle East **(**12%)	Singapore **(**40%) Thailand **(**8%) Australia **(**4.7%) China **(**3.5%) Brunei **(**3.5%) Indonesia **(**3.5%) Vietnam **(**3.5%) Japan **(**3.5%) UK **(**3.5%)
Business sector	Manufacturing **(**73%) Service **(**27%)	Manufacturing **(**91%) Service **(**9%)
Type of industries	Food and beverages **(**13%) Wood-related **(**9%) Rubber **(**7%) Chemicals and minerals **(**7%) Plastic and resin **(**6%) Palm oil **(**6%) Electric and electronics **(**5%) Automotive **(**5%) Machinery and engineering **(**5%)	Food and beverages **(**13%) Building materials **(**9%) Rubber **(**8%) Plastic and resin **(**8%) Iron and steel **(**7%) Textiles and apparel **(**6%) Automotive **(**6%)
Locations	Selangor **(**41%) Penang **(**12%) Johor **(**10%) Kuala Lumpur **(**8%) Perak **(**6%) Kedah **(**4%) Pahang **(**3%) Melaka **(**2%) Sabah **(**2%) Sarawak **(**2%)	Selangor **(**47%) Penang **(**14%) Perak **(**10%) Kuala Lumpur **(**7%) Johor **(**5%) Negeri Sembilan **(**4%)

## Measurements

We used 7-point Likert scales to measure the constructs to minimize response time and effort by the targeted respondents. We measured international performance as a second-order reflective construct of financial **(**three items) and strategic performance **(**seven items), adapted from Zhang et al. (2009) and Falahat et al. (2018). To assess digital orientation, we followed Lee and Falahat’s (2019) scale on the utilization of digital technologies for sales, search, promotion, and communication, thus covering a wider scope of digitalization instead of focusing on a specific type of digital application.

Entrepreneurial orientation was assessed using a scale developed by Jantunen et al. (2005). The scale for market orientation was adapted from Narver and Slater (1990), Jaworski and Kohli (1993), and Pelham and Wilson (1995).

Learning orientation was assessed using a scale adapted from Sok and O’Cass (2011). Commonly used earlier learning orientation scales often focus on broad learning approaches (as exemplified by Sinkula et al. 1997), while Knight et al. (2020), for example, measured international learning orientation by focusing on learning processes. We wanted to be more specific about the content of internationally oriented learning activities and thus asked about learning about foreign market rules and regulations, learning about governmental incentives, visiting foreign markets, identifying distributors, performing foreign mark risk analysis, or analyzing competitors, among others. All nine items in this scale are shown in the “Appendix” section.

We measured government support as a firm’s perceptions of the helpfulness of government support programs on a scale of 1 = unhelpful to 7 = helpful **(**Brady and Gill 1981; Grønhaug and Lorentzen 1983). Marketing capabilities were measured as first-order constructs consisting of nine items that were derived from the scale developed by Hartsfield et al. (2008). Competitive intensity and market turbulence were captured by two items, each taken from the work of Murray et al. (2011). All measurements were assessed at the level of the firm’s most important export product to its main export market.

Firm size was measured as the number of employees, and industry type was a dummy variable equaling 1 for manufacturing, 0 for service, and 2 for others (Falahat et al. 2021).

We assessed the external validity of the measurement instrument by conducting a focus group with three SME founders in Malaysia and subsequently refined it through extensive pretesting with three experts in the international business field.

We used SmartPLS version 3.2.7 **(**Ringle et al. 2015) to assess our proposed research model and to perform multigroup analysis **(**MGA) in order to compare BG and non-BG models. PLS-SEM, as an advanced multivariate approach and a nonparametric SEM technique, was deemed appropriate to perform MGA **(**Henseler et al. 2016). Prior to conducting the MGA, we examined the model’s reliability using squared standardized outer loadings and its internal consistency using composite reliability **(**CR). As shown in Table 2, with the exception of four items (i.e., LO1, GS3, GS6, MT1), all estimates well exceeded the 0.7 cutoff value for the outer loadings and CR, as recommended by Hair et al. (2010). To assess the construct validity, we examined the convergent validity using the average variance extracted **(**AVE) method. The AVE score for each construct exceeded 0.5, the threshold recommended by Bagozzi and Yi (1988).

**Table 2:** Assessment results of the measurement model

Construct/associated items	Loadings			CR		AVE	
	*Born global*		*Non-born global*	*Born global*	*Non-born global*	*Born global*	*Non-born global*
Digital orientation				0.795	0.879	0.501	0.645
DIG1	0.533		0.747				
DIG2	0.753		0.883				
DIG3	0.612		0.815				
DIG4	0.882		0.761				
Entrepreneurial orientation				0.903	0.944	0.542	0.681
EO1	0.735		0.703				
EO2	0.527		0.802				
EO3	0.801		0.904				
EO4	0.779		0.877				
EO5	0.752		0.808				
EO6	0.78		0.852				
EO7	0.675		0.8				
EO8	0.8		0.838				
Learning orientation				0.878	0.911	0.508	0.595
LO1	Dropped		Dropped				
LO2	0.703		0.703				
LO4	0.69		0.801				
LO5	0.675		0.842				
LO6	0.73		0.853				
LO7	0.661		0.75				
LO8	0.74		0.674				
LO9	0.783		0.758				
Market orientation				0.925	0.944	0.553	0.628
MO1	0.727		0.800				
MO2	0.765		0.844				
MO3	0.696		0.794				
MO4	0.708		0.713				
MO5	0.749		0.788				
MO6	0.779		0.822				
MO7	0.751		0.858				
MO8	0.677		0.651				
MO9	0.795		0.831				
MO10	0.78		0.804				
Government support				0.845	0.858	0.528	0.55
GS1	0.737		0.647				
GS2	0.885		0.813				
GS3	Dropped	Dropped					
GS4	0.761		0.719				
GS5	0.516		0.833				
GS6	Dropped		Dropped				
GS7	0.685		0.679				
Marketing capabilities				0.905	0.927	0.544	0.615
MC1	0.737		0.83				
MC2	0.778		0.735				
MC3	0.739		0.829				
MC4	0.689		0.805				
MC8	0.794		0.831				
MC9	0.771		0.751				
MC10	0.708		0.725				
MC11	0.677		0.758				
Competitive intensity				0.857	0.897	0.751	0.813
CI1	0.867		0.789				
CI2	0.935		0.938				
Market turbulence							
MT1	Dropped		Dropped				
MT2	1		1	1	1	1	1
Financial performance				0.947	0.943	0.857	0.846
FP1	0.929		0.893				
FP2	0.944		0.953				
FP3	0.905		0.912				
Strategic performance				0.937	0.956	0.681	0.758
STR_1	0.773		0.747				
STR_2	0.845		0.893				
STR_3	0.87		0.901				
STR_4	0.87		0.921				
STR_5	0.89		0.937				
STR_6	0.834		0.872				
STR_7	0.674		0.808				

For discriminant validity, we employed the heterotrait–monotrait **(**HTMT) ratio, a criterion superior to traditional assessment methods, such as cross-loadings and the Fornell–Larcker criteria **(**Henseler et al. 2015; Voorhees et al. 2016). As shown in Table 3, the HTMT ratios for all constructs were lower than 0.85, indicating adequate discriminant validity **(**Henseler et al. 2015).

**Table 3:** Discriminant validity **(**HTMT 0.85 criterion)

*Born global*										
*Constructs*	CI	DIG	EO	FIN_PRF	GOV_SUPRT	LO	MC	MO	MT	STR_PRF
CI										
DIG	0.216									
EO	0.146	0.161								
FIN_PRF	0.192	0.079	0.467							
GOV_SUPRT	0.121	0.427	0.205	0.17						
LO	0.24	0.181	0.396	0.163	0.461					
MC	0.083	0.302	0.713	0.551	0.258	0.432				
MO	0.211	0.286	0.77	0.341	0.159	0.394	0.682			
MT	0.529	0.215	0.061	0.127	0.086	0.131	0.138	0.11		
STR_PRF	0.24	0.177	0.535	0.689	0.213	0.418	0.626	0.456	0.275	
*Non-born global*										
*Constructs*	CI	DIG	EO	FIN_PRF	GOV_SUPRT	LO	MC	MO	MT	STR_PRF
CI										
DIG	0.209									
EO	0.137	0.432								
FIN_PRF	0.071	0.304	0.514							
GOV_SUPRT	0.214	0.409	0.281	0.425						
LO	0.246	0.327	0.548	0.424	0.475					
MC	0.15	0.386	0.629	0.518	0.378	0.65				
MO	0.152	0.566	0.823	0.417	0.213	0.545	0.685			
MT	0.468	0.13	0.075	0.149	0.174	0.109	0.095	0.109		
STR_PRF	0.115	0.361	0.691	0.781	0.293	0.51	0.715	0.611	0.092	

## Data analysis

Having established an adequate level of reliability and validity, we conducted an MGA to test the proposed hypotheses. A three-step process involving MICOM is employed to determine the measurement of invariance between the two groups, as follows: **(**1) conduct a configural invariance assessment; **(**2) establish the compositional invariance assessment; and **(**3) assess the equal means and variances. As recommended by Henseler et al. (2016), the least partial measurement invariance **(**first two steps) should be established when the aim of the study is to compare a model over two groups **(**BGs vs. non-BGs). Based on the MICOM guidance procedures, partial measurement invariance for BGs versus non-BGs was established for our study **(**Table 4). This indicates the acceptability of proceeding to the next step, which is to compare the differences between the two groups (Henseler et al. 2016). We used MGA PLS-SEM **(**Henseler et al. 2009) to compare the path coefficients of BG versus non-BG firms **(**as shown in Table 5).

**Table 4:** Results of invariance measurement testing using permutation

Construct	Configural invariance established **(**same algorithms for BGs)	Compositional invariance **(**correlation = 1)		Partial measurement invariance established?	Equal mean value		Equal variance		Full measurement invariance established?
		*C* = 1	Confidence interval		Differences	Confidence interval	Differences	Confidence interval	
DIG	Yes	0.946	[0.836, 1]	Yes	− 0.066	[− 0.269, 0.228]	− 0.013	[− 0.447, 0.485]	Yes
EO	Yes	0.999	[0.996, 1]	Yes	0.418	[− 0.232, 0.226]	− 0.335	[− 0.282, 0.321]	No
MO	Yes	0.999	[0.996, 1]	Yes	0.382	[− 0.245, 0.236]	− 0.209	[− 0.336, 0.36]	Yes
LO	Yes	0.991	[0.986, 1]	Yes	0.224	[− 0.226, 0.252]	− 0.302	[− 0.337, 0.342]	Yes
GOV_SUPRT	Yes	0.927	[0.876, 1]	Yes	− 0.127	[− 0.239, 0.225]	0.128	[− 0.359, 0.384]	Yes
MC	Yes	1	[0.996, 1]	Yes	0.2	[− 0.232, 0.243]	− 0.093	[− 0.289, 0.313]	Yes
CI	Yes	0.998	[0.415, 1]	Yes	0.089	[− 0.24, 0.245]	0.036	[− 0.317, 0.322]	Yes
MT	Yes	1	[1]	Yes	0.059	[− 0.233, 0.234]	0.133	[− 0.263, 0.312]	Yes
FIN_PRF	Yes	1	[1]	Yes	0.452	[− 0.228, 0.238]	0.051	[− 0.326, 0.318]	Yes
STR_PRF	Yes	1	[0.999, 1]	Yes	0.403	[− 0.241, 0.238]	− 0.328	[− 0.321, 0.313]	No
INSTRY	Yes	1	[1]	Yes	0.455	[− 0.227, 0.245]	0.855	[− 0.345, 0.384]	No
CO_SIZE	Yes	1	[1]	Yes	− 0.566	[− 0.229, 0.251]	0.137	[− 0.269, 0.291]	Yes

**Table 5:** Results of hypothesis testing

*Hypothesis*	*Relationships*	Path coefficient		*p*-values		CIs **(**bias corrected)	CIs **(**bias corrected)	Path coefficient differences	*p*-value Henseler’s MGA	Support
		*BG*	*Non-BG*	*BG*	*Non-BG*	*BG*	*Non-BG*	***(****BG3* vs*. non-BG)*	***(****BG3* vs*. non-BG)*	
*H1*	DIG—> MC	0.132	− 0.048	0.08*	0.293	[− 0.016, 0.263]	[− 0.217, 0.071]	0.18	0.074*	Yes
*H2*	EO—> MC	0.376	0.097	0***	0.239	[0.201, 0.566]	[− 0.134, 0.313]	0.279	0.053*	Yes
*H3*	LO—> MC	0.134	0.276	0.031**	0.01**	[0.011, 0.239]	[0.1, 0.492]	0.142	0.827	No
*H4*	MO—> MC	0.27	0.426	0.007***	0.001***	[0.088, 0.44]	[0.228, 0.659]	0.156	0.801	No
*H5*	GOV_SUPRT—> MC	0.058	0.175	0.228	0.031**	[− 0.125, 0.163]	[0.025, 0.329]	0.117	0.829	Yes^1^
*H6*	MC—> PRF	0.59	0.668	0***	0***	[0.468, 0.687]	[0.538, 0.764]	0.078	0.785	No
*Control 1*	CI—> PRF	− 0.19	− 0.042	0.019**	0.376	[− 0.321, − 0.022]	[− 0.252, 0.188]	0.147	0.824	
*Control 2*	MT—> PRF	− 0.055	− 0.153	0.24	0.055*	[− 0.172, 0.082]	[− 0.285, 0.014]	0.098	0.206	
*Control 3* CO_SIZE—> PRF		0.121	0.017	0.075*	0.417	[− 0.028, 0.245]	[− 0.116, 0.149]	0.104	0.181	
*Control 4* INSTRY—> PRF		0.071	− 0.171	0.219	0.083**	[− 0.084, 0.212]	[− 0.357, 0.033]	0.241	0.052*	

*CI*, confidence interval. *p < 0.1, **p < 0.05, ***p < 0.01. ^1^Hypothesis suggests no significant difference when comparing BG and non-BG firms. The results show no difference; the hypothesis is supported

We conducted an MGA to assess the structural path model to test the proposed hypotheses. The path coefficients were produced using a bootstrapping procedure with 5000 resamples **(**Hair et al. 2011). The variance inflation factor values for all of the predictor variables were below 2.65; this result indicates that there were no collinearity issues among the constructs. Table 5 presents the significant differences between BGs and non-BGs in terms of the path coefficients.

Based on the results, H1 and H2 are supported, while H3, H4, and H6 are rejected. H5, suggesting no difference between the groups, is also supported. As further discussed later, the effects of the control variable market turbulence and competition intensity are not significantly different when comparing BG and non-BGs using what are normally regarded as the most conservative techniques for group comparisons. However, we notice that the path coefficient/significance results indicate a need for more nuanced interpretation.

## Discussion

The discussion is organized with a focus on the key results and contributions of the study: **(**1) digital and entrepreneurial orientation, **(**2) governmental support orientation, **(**3) rejected hypotheses, (4) environmental conditions, and **(**5) differences between non-BGs and BGs.

### Only BG firms transform digital and entrepreneurial orientation into marketing capabilities

We included digital orientation in our model based on an expectation about its importance, particularly when considering BG firms. Moen and Rialp (2018) examined empirical studies focusing on BG firms in Europe and concluded that these firms are characterized by their focus on using information and communication technology. Our approach differs from that of most previous research, as we do not examine whether BG firms have a higher score on a digital orientation scale than non-BG firms. Instead, we focus on the following: First, within the groups of BG firms versus non-BG firms, does a stronger/weaker digital orientation translate into higher/lower marketing capabilities? Second, do these effects differ significantly between BG and non-BG firms? Indeed, the results show that digital orientation is a significant antecedent of marketing capabilities in BG firms **(**0.132, *p* < 0.1) but not in non-BG firms. Furthermore, the MGA PLS-SEM test identified statistically significant differences between the path coefficients. These results support H1 and suggest that within BG firms, higher levels of digital orientation do result in increased marketing capabilities, whereas this interaction does not exist in non-BG firms. One particular issue needs to be mentioned: the average score of BG firms in terms of digital orientation is 4.89, which is slightly lower than the average score of 5.02 among non-BGs. When we examine the items, the non-BGs do use online portals such as Alibaba and the trade platform Trademal slightly more and devote more attention to social media. This finding further underscores the need to focus on how distinct orientations may result in marketing capabilities in one group of firms but not in another group. Considering digital orientation, perhaps business model differences and the higher percentage of service sector firms in the BG group could partly explain our study results.

The starting point of our hypothesis was that digital tools may increase marketing efficiency **(**Gregory et al. 2017), improve information quality and services **(**Foroudi et al. 2017), and facilitate the internationalization of resource-constrained SMEs. Our results suggest that these effects may be evident, but not necessarily in both types of exporting firms.

Looking forward, with the rapid development of digitally driven technologies such as blockchain, the Internet of things, augmented reality, and artificial intelligence, BG firms may be expected to be better able to exploit opportunities than more traditional exporting firms. Choquette et al. (2016) described BG firms having superior performance related to turnover, employment, and market reach; the ability to transform digital orientation into capabilities may partly explain these results. Additionally, the COVID-19 pandemic may increase the importance of digital approaches to international marketing, and in such a context, BG firms may be better positioned than other international firms.

The results related to entrepreneurial orientation follow those described when examining digital orientation. There was a significant path coefficient from entrepreneurial orientation to marketing capabilities in the BG group **(**0.376, *p* < 0.01) but no such significant path when considering non-BG firms. In addition, the bootstrapping-based tests showed that the differences were statistically significant. These results support H2.

Our assessment is that this ability to transform digital and entrepreneurial orientation into capabilities may be important in explaining why BGs may successfully compete in international markets even in the face of resource constraints. Furthermore, comparing this with the observed inability of other exporting firms to transform digital and entrepreneurial orientation into marketing capabilities may notably improve our understanding of the differences between groups of exporting firms and the mechanisms influencing international performance.

### Are public support policies out of touch with the requirements of BG firms?

We expected no difference when comparing BG and non-BG firms with respect to the benefits of governmental export support initiatives. Our results show that the relationship is nonsignificant in the BG group but significant in the group of other exporting firms **(**0.175, *p* < 0.05). These results indicate a potential failure of governmental support activities if the aim is to improve the capabilities and performance of young and export growth-oriented firms. However, the MGA PLS-SEM results do not show significantly different path coefficients between the groups, and H5 was thus supported.

More specifically, the governmental support scale included a variety of initiatives and partners, exemplified by the MATRADE and financial institutions such as the export/import bank of Malaysia. If we examine the activity of MATRADE, the new exporter development program focuses on women exporters, youth exporters, and the Bumiputera geographical area. All three of these initiatives require at least 3 years of activity in Malaysia. The Mid-Tier Companies Development Programme focuses on larger, well-established firms, while the Go-Ex program requires a solid financial standing when selecting participants. Another initiative, the market development grant, excludes firms less than 1 year old. These examples suggest that the selection criteria for some of these programs in Malaysia are not perfectly aligned with the profile of BG firms. In the “Implications, limitations, and concluding remarks” section, we will further elaborate on this result, but it is an important finding that governmental support programs do not result in increased marketing capabilities for BG firms.

### The rejected hypotheses

H3 and H4 were not supported. These hypotheses posited that learning orientation and market orientation would not have a greater impact on marketing capabilities in BGs than in non-BGs. Notably, for each of these relations, the path coefficient was significant in both groups of firms. Thus, both learning orientation and market orientation do have a positive impact on marketing capabilities, and marketing capabilities do influence international performance when considering both BG firms and non-BG firms. In H6, we expected the impact of marketing capabilities on international performance to be strongest among BG firms. This hypothesis was not supported; in both groups, there was a significant relationship, but it was equal when comparing BG and non-BGs. These results indicate that we find differences (H1 and H2) and similarities (H3, H4, and H5) when trying to identify the orientations, capabilities, and performance mechanisms between the two groups. Taken together, these results suggest that firms differ in how they are able to develop marketing capabilities but not in how they use those capabilities to succeed in international markets.

### Market turbulence and competitive intensity: Indications of different effects on the two groups of firms

Our model also included two environmental control factors: competitive intensity and market turbulence. The results did not identify significant differences in the effects of these factors between the two groups of firms. Even though the path coefficient results suggest different effects, this is not supported by the MGA PLS-SEM. Nevertheless, we would like to comment on the path coefficient results, keeping in mind that this should be interpreted with more caution than most of the other results discussed.

The results related to competitive intensity among non-BGs indicate that high competition does not affect these firms systematically in a negative manner. It is possible that they have already established robust relationships within their business networks and with their customers. Another potential explanation is that because these are older firms, non-BGs may have a more solid foundation of resources to support them when facing fierce competition. While non-BGs seem able to handle intense competition, we did find a significant negative effect of this intensity on BGs’ international performance **(**− 0.16, *p* < 0.05). One possibility is that these firms have often been found to follow niche focus strategies **(**Moen and Rialp 2018), and high levels of competition intensity may indicate less successful niche strategies.

Considering market turbulence, this did not influence international performance among BGs but had a negative effect on non-BGs **(**− 0.153, *p* < 0.10). Our descriptive statistics may partly explain this result. Here, compared with non-BGs, BGs generally have stronger entrepreneurial **(**BG mean: 4.90; non-BG mean: 4.46) and market **(**BG mean: 5.30; non-BG mean: 4.92) orientations **(**see Appendix Table 6). These factors could assist BGs in better predicting the market to avoid being affected by uncertain market responses. Market turbulence also requires an ability to react and adapt, which may be easier for smaller, younger, and more innovative BG firms. Similar elements may also explain why non-BGs seem more vulnerable to market turbulence. Combined, these results indicate that BGs may be better able to handle or exploit market turbulence, while non-BGs are better suited to handle competitive intensity.

### Are BGs “a different breed” of exporting firms?

Based on empirical evidence from Australia, Rennie (1993) described BG firms as a new breed of exporters, while Cavusgil (1994) further stated as follows: “There is emerging in Australia a new breed of exporting companies, which contribute substantially to the nation’s export capital. The emergence of these exporters though not unique to the Australian economy, reflects 2 fundamental phenomena of the 1990s: 1. Small is beautiful. 2. Gradual internationalization is dead” **(**p. 18). When we examine our results, they show both similarities and differences between these two groups of firms.

Considering similarities, our results related to market orientation and learning orientation show no significant differences. Both the digital and entrepreneurial orientation path coefficients are significantly different, and the MGA PLS-SEM reveals group differences. Examining governmental support orientation, market turbulence effects, and competitive intensity effects, we observe differences when looking at significant and nonsignificant paths, but the MGA PLS-SEM does not show these path coefficients to significantly differ across the two groups. We further notice that the BG model explains 54.4% of the variance **(***R*^2^) for marketing capabilities and 61.3% of the variance **(***R*^2^) for international performance. For the non-BG model, the model explains 61.5% of the variance in marketing capabilities, and for international performance, it explains 69.2%. These results indicate that it may be more difficult to explain variation in both international performance and marketing capabilities in BG firms than in non-BG firms.

Regarding whether BG firms represent a new type or breed of exporters, our study suggests that important differences exist in how these firms transform manager orientations into marketing capabilities and how they are influenced by environmental factors compared to other exporting firms. Based on empirical data from Norway, Denmark, and France, Moen and Servais (2002) concluded, “It seems that the future export involvement of the firm is influenced to a large extent by its behavior shortly after establishment” **(**p. 69). Our results go even further, showing that BG firms not only develop differently than other exporting firms, but also differ from those firms in the mechanisms driving their development. While Morgan et al. (2018) called for studies focusing on differences in antecedents of marketing capabilities across international stages, our results suggest that it may be even more important to investigate groups of exporting firms in which there are differences in how manager orientations influence capability development and differences in their ability to handle variations in environmental conditions such as market turbulence and competitive intensity.

Hennart (2014) suggested that the business model of INVs/BG is an important factor explaining their international growth. If we look at our dataset, we notice differences in the types of industries (more advanced industries among BG firms and a higher percentage of services), indicating differences in what they sell and customer groups. How they sell could also be different, not least related to digital elements. In their empirical study, Martin et al. (2017) concluded that what they described as positional advantage was important for understanding the performance of BG firms. Our results could be interpreted as supporting Hennart (2014), where the combined effects of industrial types, customer groups, and business models do exist and differentiate the two groups of firms.

Wikipedia defines a breed as a specific group of animals having homogeneous appearance and behavior and/or other characteristics that distinguish it from other organisms of the same species **(**https://en.wikipedia.org/wiki/Breed). We are confident that the findings show substantial evidence that the antecedents to international performance and to BG performance are not interchangeable under certain circumstances. Even though some firms have characteristics at the intersection between BGs and non-BGs (Kuivalainen et al. 2012; Vissak and Masso 2014), our results suggest that this group division makes sense. Thus, our results could be interpreted as supporting BGs as a new breed of firms **(**Rennie 1993). The critical notion is that dissimilar antecedents for the same type of capabilities could lead to different outcomes depending on whether a firm is a BG or a non-BG. Our study results could help managers and policymakers devise strategies to enhance the international performance of both types of firms.

## Implications, limitations, and concluding remarks

Marketing capabilities play an essential role in the achievement of international performance. The notion that firms take different routes to develop their marketing capabilities can help international business scholars and managers to better interpret the findings in the existing literature based on the specific research setting. In the following sections, we comment on the implications of our study, organizing this discussion according to the implications for public policy, for managers and for research.

### Implication for public policy

Policymakers aiming to encourage firms to engage in or increase their export sales may assume that both types of firms are interchangeable, and this assumption could potentially lead to different outcomes than their intended purpose. Our results suggest that the government in Malaysia should review the contents and qualification criteria of existing government support programs, as these programs currently do not significantly assist BGs in improving their marketing capabilities. Most are designed for experienced firms; consequently, young firms with limited financial histories and a lack of export experience may be unable to reap the benefits of these programs.

It is crucial for policymakers to understand how they may better plan and develop government support programs that are customized to the needs of their intended target groups. Our findings on the relationship between government support and marketing capabilities reveal an important perspective for scholars and policymakers: how does the impact of government support programs vary between BGs and non-BGs? In the development of export promotion initiatives, three questions should be kept in mind: Do non-BG firms have more capacity to participate and learn from such programs, while resource constraints limit this effect in BG firms? Does the content of governmental export support initiatives fit the needs of non-BG firms better than the needs of BG firms? Does there exist some type of systematic recruitment/selection effect regarding firm participation in governmental support programs where non-BGs seem to better fit the design and recruitment processes?

### Implications for managers

The empirical results suggest that international business and marketing scholars should test the possibility that different antecedents have different influences on BG versus non-BG firms, particularly if digital and entrepreneurial orientations are included as predictors.

Strong digital and entrepreneurial orientations do not translate into marketing capabilities for non-BG firms. This result implies that instead of focusing only on the development of these firms’ digital and entrepreneurial orientations, managers should focus on how these firms can take advantage of these orientations. Managers may consider whether firms’ market and learning orientations interfere with these firms’ marketing capabilities, as the non-BG group was found to have lower means for learning orientation.

Considering environmental factors, Efrat and Shoham (2012) did find that market turbulence reduces the probability of other players becoming new entrants into the market and prevents imitation and fierce price wars between competitors, which allow innovative firms **(**as most BGs are) to maximize the benefits of their unique offerings. Guo et al. (2018) also provided a possible explanation for marketing capabilities that are more dynamic in nature and help firms survive under conditions of either high or low turbulence. In this comparison case, BGs’ marketing capabilities are probably more dynamic than those of non-BGs. Understanding how these factors represent weaknesses or strengths is important for managers, who should attempt to reduce such effects or, if possible, avoid environmental conditions that are most challenging for their type of firm.

The different mechanisms between the antecedents of marketing capabilities for BG versus non-BG firms may also partly explain why not every firm is able to pursue a rapid and early internationalization process. A key implication of our study is that the two groups of firms are not equal; if they are managed similarly, misjudgment will occur.

### Implications for research

In emerging markets, we have a limited understanding of whether and how environmental factors, such as competitive intensity and market turbulence, affect international performance (Ismail and Kuivalainen 2015). In our study, however, we chose to explore the direct influence of these factors to provide different insights. Our attempts to show these relationships provide guidance for future studies on the application of these variables in similar contexts. The analysis shows that the negative effect of competitive intensity is insignificant for non-BG firms and that the negative effect of market turbulence is insignificant for BG firms. Thus, future research may consider setting competitive intensity and market turbulence as control variables, similar to the model that Kaleka (2012) tested.

Furthermore, our study reveals positive relationships between learning, market orientation, and marketing capabilities for both BG and non-BG firms. Future research may relate these strategic orientations to different types of capabilities for both firm types. Scholars may also conduct research on all types of exporters and divide their samples into BG and non-BG firms only when further investigation is necessary to better interpret the findings.

Our operational definition of BG firms is based on the time until they start exporting (3 years); the BG group on average had an export share of 75%, compared to 12% in the non-BG group. In principle, there could be firms in the BG group with low export shares and firms in the non-BG group with high export shares. However, based on the average scores, this would be an exception, and we do not expect the way we operationalize the BG concept to significantly influence our results. It may also be questioned whether our two groups just represent firms in different stages of internationalization. However, we regard it as unlikely that young firms with early internationalization and high export shares would develop into firms with limited international involvement as they grow older. Nevertheless, we have treated both groups as homogenous and not tried to classify them on the basis of internationalization stages. There may still exist substantial differences within the groups, and a variety of international stages or pathways could exist. In further studies, attention should be given to nuanced classifications of groups of firms in order to advance our understanding of similarities and differences between subgroups.

Scholars may further investigate the different issues and topics related to governmental export promotion programs, such as comparing their impact on BG versus non-BG firms in terms of their perceived usefulness, adoption rate, and effectiveness. It is also worth further investigating why government support programs do not translate into marketing capabilities for BGs. We conjecture that the contents and qualification criteria of these programs play a role here. This topic would be interesting to further explore through the lens of institutional research.

## Limitations

Our single-country setting limits the generalizability of our study findings. Specifically, the contents of government support programs may vary from one country to another. Thus, future research may validate the direction and magnitude of the antecedent–capability–performance relationships for BGs and non-BGs in different regions or countries. Second, the cross-sectional nature of the study limits the understanding of how these relationships evolve during the internationalization process. A longitudinal study could provide essential insights into how changes in the antecedents of marketing capabilities and their impacts evolve across different stages of a firm’s internationalization process. Third, in assessing all constructs, we asked respondents to focus on the export product most important to its main export market when answering the questions. Although this approach is more often used in empirical studies, it may influence the results, and future research may address this limitation.

## Appendix

**Table 6:** Descriptive analysis, means, and standard deviations

	*Born global*		*Non-born global*	
Construct/associated items	Mean	Std. deviation	Mean	Std. deviation
***Digital orientation***	**4.89**	**0.366**	**5.02**	**0.291**
DIG1 **(**online B2B portals, e.g., Alibaba.com, Trademal.com, C21.com)	4.21	1.980	4.37	1.782
DIG2 **(**search engine, e.g., Google.com)	5.44	1.499	5.37	1.423
DIG3 **(**social network, e.g., Facebook.com, Instagram, LinkedIn.com)	3.88	1.882	4.26	1.687
DIG4 **(**advance communication, e.g., e-mail, online chat, VOIP)	6.06	1.183	6.07	1.135
***Entrepreneur orientation***	**4.90**	**0.099**	**4.46**	**0.105**
EO1 **(**progressive and innovative production)	4.60	1.383	4.00	1.495
EO2 **(**risk-associated projects)	4.38	1.471	4.17	1.416
EO3 **(**best practices adoption)	5.24	1.246	4.52	1.412
EO4 **(**exploitation of new practices)	4.90	1.381	4.44	1.492
EO5 **(**recognition of technological changes)	5.34	1.308	4.95	1.336
EO6 **(**adaptability to sudden change)	4.93	1.147	4.72	1.224
EO7 **(**bold actions in the face of uncertainty)	4.85	1.291	4.38	1.520
EO8 **(**resource allocation to new areas)	4.94	1.363	4.51	1.299
***Learning orientation***	**4.94**	**0.178**	**4.65**	**0.140**
LO1 **(**study foreign market rules and regulations **(**e.g., customs, import tax)	4.83	1.544	4.49	1.646
LO2 **(**learn about government incentives)	4.74	1.531	4.51	1.555
LO3 **(**visit to foreign market)	4.91	1.596	4.29	1.622
LO4 **(**prepare **(**country) risk analysis)	4.21	1.540	3.95	1.630
LO5 **(**domestic–international coordination)	4.77	1.451	4.51	1.387
LO6 **(**learn foreign business culture **(**e.g., negotiation))	4.66	1.448	4.56	1.576
LO7 **(**identify qualified international distributors or agents **(**international channel members))	5.06	1.293	4.78	1.417
LO8 **(**identify customer requirements)	5.93	1.025	5.71	1.226
LO9 **(**identify competitor strengths/weaknesses and strategies)	5.34	1.342	5.02	1.511
***Market orientation***	**5.30**	**0.154**	**4.92**	**0.081**
MO1 **(**communicating customer experience)	5.13	1.321	4.52	1.352
MO2 **(**contribution to creating value)	5.15	1.470	4.76	1.411
MO3 **(**discussion of competitor**(**s))	5.09	1.411	4.58	1.426
MO4 **(**launch of intensive campaigns)	5.05	1.323	4.57	1.443
MO5 **(**various business functions **(**marketing/sales, manufacturing, finance/R&D) are integrated in serving the market)	5.39	1.297	5.01	1.384
MO6 **(**understanding customer needs as competitive advantage)	5.69	1.047	5.35	1.290
MO7 **(**value creation–driven strategy)	5.61	1.088	5.30	1.218
MO8 **(**systematic assessment of customer satisfaction)	5.15	1.497	4.94	1.375
MO9 **(**close attention to aftersales service)	5.47	1.276	5.24	1.246
MO10 **(**quick customer response)	5.64	1.171	5.20	1.254
***Government support***	**4.76**	**0.113**	**4.99**	**0.160**
GS1 **(**Malaysia External Trade Development Corporation **(**MATRADE))	5.51	1.585	5.63	1.572
GS2 **(**SME Corporation Malaysia **(**SME Corp))	4.77	1.848	4.96	1.840
GS3 **(**Standard and Industrial Research Institute of Malaysia **(**SIRIM))	4.52	1.732	5.08	1.545
GS4 **(**Malaysian Technology Corporation **(**MTDC))	3.82	1.844	3.94	1.674
GS5 **(**Chambers of commerce **(**MICCI)	4.73	1.799	4.91	1.604
GS6 **(**Federation of Malaysian Manufactures **(**FMM))	5.14	1.767	5.34	1.305
GS7 **(**financial institutions **(**e.g., EXIM Bank, SME bank, MIDF))	4.82	1.946	5.06	1.588
***Marketing capabilities***	**5.10**	**0.127**	**4.94**	**0.086**
MC1 **(**monitoring and evaluation of marketing activities)	5.06	1.236	4.78	1.192
MC2 **(**ability to segment and target specific markets)	5.20	1.115	4.87	1.093
MC3 **(**intimate knowledge of customers’ preferences)	5.36	1.090	5.15	1.101
MC4 **(**effectiveness of distribution networks)	5.00	1.157	4.83	1.210
MC8 **(**ability to use marketing tools)	4.95	1.374	4.92	1.294
MC9 **(**creating products/services uniqueness)	5.01	1.378	5.07	1.281
MC10 **(**advertising and promotion effectiveness)	4.68	1.334	4.55	1.262
MC11 **(**company image)	5.57	1.096	5.29	1.177
***Competitive intensity***	**5.01**	**0.039**	**4.88**	**0.136**
CI1 **(**cutthroat competition)	4.97	1.501	4.79	1.610
CI2 **(**easily matchable by competitors)	5.06	1.446	4.97	1.418
***Market turbulence***	**4.56**	**0.023**	**4.54**	**0.046**
MT1 **(**customers look for new products all the time)	4.59	1.648	4.62	1.566
MT2 **(**customers look for new suppliers all the time)	4.52	1.616	4.46	1.500
***Financial performance***	**4.88**	**0.149**	**4.29**	**0.169**
FP1 **(**profits from export sales)	4.84	1.275	4.30	1.247
FP 2 **(**export sales growth)	4.90	1.320	4.41	1.278
FP 3 **(**contribution of export sales to total sales)	4.89	1.552	4.15	1.553
***Strategic performance***	**5.06**	**0.074**	**4.59**	**0.083**
STR_1 **(**relationship with agent/distributor)	5.22	1.286	4.84	1.370
STR_2 **(**expanding market coverage)	5.12	1.325	4.56	1.476
STR_3 **(**entering new market segments)	4.81	1.351	4.26	1.528
STR_4 **(**increasing awareness)	5.02	1.292	4.50	1.437
STR_5 **(**establishing product presence)	5.00	1.327	4.51	1.485
STR_6 **(**improving knowledge of international markets)	5.17	1.262	4.81	1.435
STR_7 **(**increasing speed of customers’ product acceptance)	5.00	1.241	4.59	1.434
STR_8 **(**rate of company success)	5.17	1.116	4.67	1.257
